# Effectiveness of Bivalent mRNA Vaccines in Preventing Symptomatic SARS-CoV-2 Infection — Increasing Community Access to Testing Program, United States, September–November 2022

**DOI:** 10.15585/mmwr.mm7148e1

**Published:** 2022-12-02

**Authors:** Ruth Link-Gelles, Allison Avrich Ciesla, Katherine E. Fleming-Dutra, Zachary R. Smith, Amadea Britton, Ryan E. Wiegand, Joseph D. Miller, Emma K. Accorsi, Stephanie J. Schrag, Jennifer R. Verani, Nong Shang, Gordana Derado, Tamara Pilishvili

**Affiliations:** ^1^National Center for Immunization and Respiratory Diseases, CDC; ^2^Eagle Health Analytics, San Antonio, Texas; ^3^Center for Preparedness and Response, CDC; ^4^Epidemic Intelligence Service, CDC.

On September 1, 2022, bivalent COVID-19 mRNA vaccines, composed of components from the SARS-CoV-2 ancestral and Omicron BA.4/BA.5 strains, were recommended by the Advisory Committee on Immunization Practices (ACIP) to address reduced effectiveness of COVID-19 monovalent vaccines during SARS-CoV-2 Omicron variant predominance (*1*). Initial recommendations included persons aged ≥12 years (Pfizer-BioNTech) and ≥18 years (Moderna) who had completed at least a primary series of any Food and Drug Administration–authorized or –approved monovalent vaccine ≥2 months earlier (*1*). On October 12, 2022, the recommendation was expanded to include children aged 5–11 years. At the time of recommendation, immunogenicity data were available from clinical trials of bivalent vaccines composed of ancestral and Omicron BA.1 strains; however, no clinical efficacy data were available. In this study, effectiveness of the bivalent (Omicron BA.4/BA.5–containing) booster formulation against symptomatic SARS-CoV-2 infection was examined using data from the Increasing Community Access to Testing (ICATT) national SARS-CoV-2 testing program.* During September 14–November 11, 2022, a total of 360,626 nucleic acid amplification tests (NAATs) performed at 9,995 retail pharmacies for adults aged ≥18 years, who reported symptoms consistent with COVID-19 at the time of testing and no immunocompromising conditions, were included in the analysis. Relative vaccine effectiveness (rVE) of a bivalent booster dose compared with that of ≥2 monovalent vaccine doses among persons for whom 2–3 months and ≥8 months had elapsed since last monovalent dose was 30% and 56% among persons aged 18–49 years, 31% and 48% among persons aged 50–64 years, and 28% and 43% among persons aged ≥65 years, respectively. Bivalent mRNA booster doses provide additional protection against symptomatic SARS-CoV-2 in immunocompetent persons who previously received monovalent vaccine only, with relative benefits increasing with time since receipt of the most recent monovalent vaccine dose. Staying up to date with COVID-19 vaccination, including getting a bivalent booster dose when eligible, is critical to maximizing protection against COVID-19 (*1*).

The ICATT program was designed to increase access to COVID-19 testing in areas with high social vulnerability^†^ through contracts with retail pharmacy chains to provide SARS-CoV-2 testing at no cost to the recipient at selected sites nationwide (*2*). ICATT vaccine effectiveness (VE) methods have been described previously (*3*). Briefly, at test registration, adults report their vaccination history^§^ and information on current COVID-19 symptoms, previous SARS-CoV-2 infection, and underlying medical conditions. Adults receiving testing at participating sites during September 14–November 11, 2022, (when Omicron variant BA.4/BA.5 lineages and their sublineages predominated^¶^) who reported one or more COVID-19–compatible symptoms were included; case-patients were persons who received a positive rapid or laboratory-based NAAT result; control-patients were those who received a negative NAAT result. Tests from persons who reported an immunocompromising condition (*4*), who received non-mRNA COVID-19 vaccines, who had received only a single monovalent mRNA vaccine dose or >4 monovalent mRNA doses, or who had received their last monovalent dose <2 months before the SARS-CoV-2 test were excluded from analyses.** In addition, tests from persons who reported a positive result during the preceding 90 days^††^ were excluded to avoid analyzing repeated tests for the same illness episode or reinfections within a relatively short time frame. Absolute VE (aVE) was calculated by comparing the odds of receipt of a bivalent booster dose (after 2, 3, or 4 monovalent vaccine doses) to being unvaccinated (zero doses of any COVID-19 vaccine) among case- and control-patients. rVE was calculated by comparing the odds of receiving a bivalent booster dose (after 2, 3, or 4 monovalent doses) versus not receiving a bivalent booster dose (but receiving 2, 3, or 4 monovalent doses). To explore how waning of protection after receipt of the most recent monovalent vaccine dose influenced the measured relative effectiveness of a subsequent bivalent booster dose, rVE of a bivalent booster dose was calculated by interval since receipt of the most recent monovalent vaccine dose among those who had not received a bivalent booster (2–3 months, 4–5 months, 6–7 months, and ≥8 months). Odds ratios (ORs) were calculated using multivariable logistic regression^§§^; VE was calculated as (1 − OR) x 100. Analyses were conducted using R software (version 4.1.2; R Foundation). This activity was reviewed by CDC and was conducted consistent with applicable federal law and CDC policy.^¶¶^

Among persons aged ≥18 years reporting COVID-19–compatible symptoms, 360,626 tests were included; of these, 121,687 (34%) persons received positive test results (Table 1). Among these case-patients, 28,874 (24%) reported being unvaccinated, 87,013 (72%) had received 2, 3, or 4 monovalent vaccine doses but no bivalent booster dose, and 5,800 (5%) had received a bivalent booster dose. Among 238,939 control-patients who received negative test results, 72,010 (30%) reported being unvaccinated, 150,455 (63%) had received 2, 3, or 4 monovalent vaccine doses but no bivalent booster dose, and 16,474 (7%) had received a bivalent booster dose. Median interval between receipt of the bivalent booster dose and SARS-CoV-2 testing was 1 month (range = 0–2 months) and did not vary by case status. Self-reported infection >90 days before the current test was more common among persons who received a negative test result (43%) than among those who received a positive test result (22%).

**TABLE 1:** Characteristics of patients with SARS-CoV-2 tests conducted at national pharmacy testing program locations (N = 360,626) — Increasing Community Access to Testing program, United States, September–November 2022

Characteristic	SARS-CoV-2 test result (col. %)		Number and type of mRNA COVID-19 vaccine doses received* before test date, no. (row %)					
	Positive (case-patients)	Negative (control-patients)	Unvaccinated	2 monovalent doses	3 monovalent doses	4 monovalent doses^†^	≥2 monovalent doses	≥2 monovalent plus bivalent booster
**SARS-CoV-2 status** ^§^								
Positive (case-patients)	121,687 (100)	0 (—)	28,874 (24)	36,429 (30)	41,409 (34)	9,175 (8)	87,013 (72)	5,800 (5)
Negative (control-patients)	0 (—)	238,939 (100)	72,010 (30)	72,352 (30)	65,122 (27)	12,981 (5)	150,455 (63)	16,474 (7)
**Time frame of test**								
Sep 14–Oct 29, 2022	98,729 (81)	194,150 (81)	81,876 (28)	88,392 (30)	88,768 (30)	19,425 (7)	196,585 (67)	14,418 (5)
Oct 30–Nov 11, 2022	22,958 (19)	44,789 (19)	19,008 (28)	20,389 (30)	17,763 (26)	2,731 (4)	40,883 (60)	7,856 (12)
**Age group, yrs**								
18–49	75,012 (62)	171,125 (72)	81,296 (33)	82,488 (34)	71,881 (29)	0 (—)	154,369 (63)	10,472 (4)
50–64	29,896 (25)	43,179 (18)	14,366 (20)	19,688 (27)	22,580 (31)	11,055 (15)	53,323 (73)	5,386 (7)
≥65	16,779 (14)	24,635 (10)	5,222 (13)	6,605 (16)	12,070 (29)	11,101 (27)	29,776 (72)	6,416 (15)
**Sex**								
Female	68,487 (56)	150,790 (63)	57,988 (26)	66,662 (30)	66,983 (31)	13,661 (6)	147,306 (67)	13,983 (6)
Male	53,029 (44)	87,644 (37)	42,818 (30)	41,915 (30)	39,245 (28)	8,486 (6)	89,646 (64)	8,209 (6)
Other	171 (0.1)	505 (0.2)	78 (12)	204 (30)	303 (45)	9 (1)	516 (76)	82 (12)
**Race and ethnicity**								
Black or African American, non-Hispanic	15,881 (13)	39,592 (17)	20,759 (37)	19,729 (36)	11,190 (20)	2,321 (4)	33,240 (60)	1,474 (3)
Hispanic or Latino	22,694 (19)	48,109 (20)	22,074 (31)	25,281 (36)	19,408 (27)	2,141 (3)	46,830 (66)	1,899 (3)
Other, non-Hispanic	14,583 (12)	25,453 (11)	7,796 (19)	10,552 (26)	16,811 (42)	2,240 (6)	29,603 (74)	2,637 (7)
White, non-Hispanic	60,315 (50)	110,191 (46)	40,756 (24)	46,158 (27)	53,483 (31)	14,654 (9)	114,295 (67)	15,455 (9)
Unknown	8,214 (7)	15,594 (7)	9,499 (40)	7,061 (30)	5,639 (24)	800 (3)	13,500 (57)	809 (3)
**HHS testing site region** ^¶^								
Region 1	8,705 (7)	15,181 (6)	5,088 (21)	5,653 (24)	9,005 (38)	1,943 (8)	16,601 (70)	2,197 (9)
Region 2	13,533 (11)	19,672 (8)	7,698 (23)	8,918 (27)	12,151 (37)	2,199 (7)	23,268 (70)	2,239 (7)
Region 3	9,802 (8)	17,519 (7)	7,090 (26)	7,618 (28)	8,564 (31)	1,957 (7)	18,139 (66)	2,092 (8)
Region 4	24,059 (20)	57,781 (24)	28,092 (34)	26,615 (33)	18,942 (23)	4,525 (6)	50,082 (61)	3,666 (4)
Region 5	25,382 (21)	44,689 (19)	19,072 (27)	20,873 (30)	20,740 (30)	4,403 (6)	46,016 (66)	4,983 (7)
Region 6	12,601 (10)	31,708 (13)	14,127 (32)	15,290 (35)	10,892 (25)	2,140 (5)	28,322 (64)	1,860 (4)
Region 7	3,451 (3)	6,715 (3)	3,004 (30)	3,318 (33)	2,735 (27)	537 (5)	6,590 (65)	572 (6)
Region 8	3,060 (3)	5,423 (2)	1,485 (18)	2,861 (34)	2,973 (35)	527 (6)	6,361 (75)	637 (8)
Region 9	18,771 (15)	35,126 (15)	14,080 (26)	15,321 (28)	17,755 (33)	3,433 (6)	36,509 (68)	3,308 (6)
Region 10	2,323 (2)	5,125 (2)	1,148 (15)	2,314 (31)	2,774 (37)	492 (7)	5,580 (75)	720 (10)
SVI,** **mean (SD)**	0.5 (0.3)	0.5 (0.3)	0.6 (0.3)	0.5 (0.3)	0.5 (0.3)	0.5 (0.3)	0.5 (0.3)	0.5 (0.3)
**History of self-reported SARS-CoV-2 positive test result**								
None	95,378 (78)	136,420 (57)	59,380 (26)	63,497 (27)	73,538 (32)	18,420 (8)	155,455 (67)	16,963 (7)
Positive >90 days before current test	26,309 (22)	102,519 (43)	41,504 (32)	45,284 (35)	32,993 (26)	3,736 (3)	82,013 (64)	5,311 (4)
**SARS-CoV-2 test type**								
Rapid NAAT^††^	39,729 (33)	84,511 (35)	33,055 (27)	44,280 (36)	34,218 (28)	6,281 (5)	84,779 (68)	6,406 (5)
Laboratory-based NAAT^§§^	81,958 (67)	154,428 (65)	67,829 (29)	64,501 (27)	72,313 (31)	15,875 (7)	152,689 (65)	15,868 (7)
**Self-reported one or more chronic underlying condition** ^¶¶^								
No	94,236 (77)	187,842 (79)	85,207 (30)	86,234 (31)	81,463 (29)	13,581 (5)	181,278 (64)	15,593 (6)
Yes	27,451 (23)	51,097 (21)	15,677 (20)	22,547 (29)	25,068 (32)	8,575 (11)	56,190 (72)	6,681 (9)
**For persons who received only monovalent mRNA doses, no. of mos since most recent dose**								
2–3	3,718 (3)	7,540 (3)	0 (—)	1,966 (17)	3,446 (31)	5,846 (52)	11,258 (100)	0 (—)
4–5	7,188 (6)	12,284 (6)	0 (—)	2,907 (15)	5,517 (28)	11,048 (57)	19,472 (100)	0 (—)
6–7	6,110 (5)	11,396 (5)	0 (—)	4,002 (23)	9,061 (52)	4,443 (25)	17,506 (100)	0 (—)
≥8	69,592 (60)	118,304 (53)	0 (—)	99,906 (53)	87,943 (47)	47 (0.03)	187,896 (100)	0 (—)

**Abbreviations:** HHS = U.S. Department of Health and Human Services; ICATT = Increasing Community Access to Testing program; NAAT = nucleic acid amplification test; SVI = social vulnerability index.

* Only month and year of receipt were reported for each vaccination dose from some participating pharmacies; therefore, the number of months between a vaccine dose and testing is a whole number calculated as the difference between the month and year of testing and the month and year of the vaccine dose. Persons reporting an mRNA booster dose on or after September 1, 2022, were assumed to have received a bivalent dose because no monovalent mRNA doses were authorized for use as booster doses at that time. For doses received in the same month or the month before SARS-CoV-2 testing, an additional question was asked to specify whether the dose was received ≥2 weeks before testing, and only doses received ≥2 weeks before testing were included.

^†^ Persons aged <50 years without moderate or severe immunocompromise were not eligible for a fourth monovalent (second booster) dose. Because of timing of authorization, not enough persons ≥8 months from the fourth dose (second monovalent booster) were available to include in analyses.

^§^ SARS-CoV-2 status after the most recent vaccine dose received.

^¶^ Regions defined by HHS and include only states and territories with ICATT sites. U.S. Virgin Islands (Region 2) and Federated States of Micronesia, Guam, Marshall Islands, Northern Mariana Islands, Palau, and American Samoa (Region 9) were not included because they did not have pharmacies participating in ICATT. https://www.hhs.gov/about/agencies/iea/regional-offices/index.html

** SVI is a tool that uses U.S. Census Bureau data on 16 social factors to rank social vulnerability by U.S. Census Bureau tract. The scale is from 0 to 1; higher SVIs represent more vulnerable communities. Tests with missing SVI data (<1% of total) were excluded from all analyses. https://www.atsdr.cdc.gov/placeandhealth/svi/data_documentation_download.html

^††^ Rapid NAAT was performed on-site on self-collected nasal swabs using ID Now (Abbott Diagnostics Scarborough Inc.) and Accula (Thermo Fisher Scientific).

^§§^ Laboratory-based NAAT was performed on self-collected nasal swabs at contracted laboratories using a variety of testing platforms.

^¶¶^ Underlying conditions included on the survey were heart conditions, high blood pressure, overweight or obesity, diabetes, current or former smoker, kidney failure or end stage renal disease, cirrhosis of the liver, chronic lung disease (such as chronic obstructive pulmonary disease, moderate to severe asthma, cystic fibrosis, or pulmonary embolism).

aVE of a bivalent booster dose received after ≥2 monovalent doses (compared with being unvaccinated) was similar among persons aged 50–64 years (28%) and ≥65 years (22%) but varied somewhat by number of previous monovalent vaccine doses (Table 2). Among adults aged 18–49 years, aVE after ≥2 monovalent doses (43%) was higher than that for older age groups and did not vary among those who received 2 or 3 previous monovalent vaccine doses.

**TABLE 2:** Absolute vaccine effectiveness against symptomatic SARS-CoV-2 infection for a single bivalent mRNA COVID-19 booster dose received after 2, 3, or 4 doses of monovalent vaccine compared with no doses, by age group and number of monovalent COVID-19 vaccine doses — Increasing Community Access to Testing program, United States, September–November 2022

Age group, yrs	Absolute VE (95% CI), by no. of monovalent doses received before the bivalent vaccine dose			
	2 doses	3 doses	4 doses*	≥2 doses
**18–49**	41 (31–49)	43 (39–46)	NA	43 (39–46)
**50–64**	50 (35–61)	25 (17–33)	28 (20–34)	28 (22–33)
**≥65**	32 (9–49)	19 (8–29)	23 (15–30)	22 (15–29)

**Abbreviations:** NA = not applicable; VE = vaccine effectiveness.

* Persons aged <50 years without moderate or severe immunocompromise were not eligible for a fourth monovalent (second booster) dose.

Among persons who received ≥2 monovalent vaccine doses, rVE increased with time since the most recent monovalent vaccine dose in all age groups (Table 3). At 2–3 months and ≥8 months after receipt of the most recent monovalent dose, rVE of a bivalent mRNA COVID-19 vaccine dose was 30% and 56% among persons aged 18–49 years, 31% and 48% among persons aged 50–64 years, and 28% and 43% among persons aged ≥65 years, respectively.

**TABLE 3:** Relative vaccine effectiveness of a single bivalent mRNA COVID-19 booster dose against symptomatic SARS-CoV-2 infection* received after 2, 3, or 4 monovalent vaccine doses, by age group, number of monovalent COVID-19 vaccine doses received, and interval since last monovalent dose — Increasing Community Access to Testing program, United States, September–November 2022

Age group, yrs/mos since receipt of most recent monovalent dose	Relative VE (95% CI), by no. of monovalent doses received^†^			
	2 doses	3 doses	4 doses^§^	≥2 doses
**18–49**				
2–3	45 (31–56)	24 (14–33)	NA	30 (22–37)
4–5	47 (35–57)	41 (35–47)	NA	43 (38–48)
6–7	42 (30–52)	47 (42–52)	NA	46 (41–50)
≥8	53 (45–60)	58 (56–61)	NA	56 (53–58)
**50–64**				
2–3	—	15 (–4–31)	33 (24–41)	31 (24–38)
4–5	44 (18–62)	31 (18–42)	36 (29–43)	36 (30–41)
6–7	46 (22–62)	36 (25–45)	40 (32–47)	38 (32–43)
≥8	61 (49–70)	51 (45–55)	NA	48 (45–51)
**≥65**				
2–3	—	—	32 (23–40)	28 (19–35)
4–5	—	21 (1–36)	36 (29–42)	33 (27–39)
6–7	—	14 (–6–30)	40 (33–46)	36 (29–41)
≥8	45 (27–58)	42 (35–48)	NA	43 (39–46)

**Abbreviations:** NA = not applicable; VE = vaccine effectiveness.

* VE estimates with 95% CIs >50 percentage points are not shown because of imprecision.

^†^ Total number of monovalent doses received for persons who did and did not receive a bivalent booster dose.

^§^ Persons aged <50 years without moderate or severe immunocompromise were not eligible for a fourth monovalent (second booster) dose. Because of timing of authorization, not enough persons ≥8 months from the fourth dose (second booster) were available to include in analyses.

## Discussion

Among symptomatic adults who received testing for SARS-CoV-2 infection at pharmacies nationwide during September 14–November 11, 2022, bivalent mRNA vaccines provided additional protection against infection compared with previous vaccination with 2, 3, or 4 monovalent vaccines alone. These are the first published estimates of VE for newly authorized bivalent mRNA booster vaccines. In this study, relative benefits of a bivalent booster compared with monovalent vaccine doses alone increased with time since receipt of last monovalent dose.

Postauthorization immunogenicity studies have shown similar neutralizing antibody titers to BA.4/BA.5 after receipt of either a monovalent or BA.4/BA.5–containing bivalent vaccine as a fourth dose (*5*,*6*); however, immunogenicity studies are not generally designed to measure clinical impact. Findings from this real-world VE study indicate that the bivalent formulations authorized in the United States provide additional protection when administered to persons who previously received 2, 3, or 4 doses of monovalent mRNA vaccines.

Waning VE with time since monovalent vaccine receipt has been observed during the Omicron-predominant period, with more rapid waning during the period when Omicron BA.4/BA.5 lineages predominated.*** Results from this study show that bivalent boosters provide protection against symptomatic SARS-CoV-2 infection during circulation of BA.4/BA.5 and their sublineages and restore protection observed to wane after monovalent vaccine receipt, as demonstrated by increased rVE with longer time since the most recent monovalent dose. Most tests (81%) in this study were conducted during a period of BA.4/BA.5 predominance. Results limited to the period of BA.4/BA.5 predominance were not meaningfully different from the results shown, which include data from the period when BA.4/BA.5 sublineages (including BA.4.6, BA.5.2.6, BF.7, BQ.1, and BQ.1.1) predominated.

This study evaluated aVE and rVE by number of previous monovalent doses received and generally found similar additional benefit of the bivalent vaccine regardless of the number of previous monovalent vaccine doses received, when controlling for time since receipt of the last monovalent dose. These findings support the current COVID-19 vaccination policy recommending a bivalent booster dose for adults who have completed at least a primary mRNA vaccination series, irrespective of the number of monovalent doses previously received.

In the United States, >90% of adults have received ≥1 COVID-19 vaccine dose.^†††^ Therefore, aVE should be interpreted with caution because unvaccinated persons might have different behaviors or a fundamentally different risk for acquiring COVID-19 compared with vaccinated persons. aVE in this study appeared lower in persons aged ≥50 years who received 3 or 4 monovalent doses before a bivalent booster dose compared with those who received only 2 monovalent doses before a bivalent booster dose; this might be because of differential rates of previous infection or differences in behaviors in those who had not previously received a booster dose compared with those who remained up to date with previous booster dose recommendations.

The findings in this study are subject to at least six limitations. First, vaccination status, previous infection history, and underlying medical conditions were self-reported and might be subject to recall bias. In particular, if previous infection provides protection against repeat infection, then VE estimates in this study would likely be biased toward the null, because self-reported previous infection differed by vaccination status, and statistical power was not sufficient to stratify VE estimates by presence of previous infection. In addition, previous infection might have been underreported (*7*). Second, acceptance of bivalent booster doses to date has been low (approximately 10% of persons aged ≥5 years as of November 15, 2022),^§§§^ which could bias the results if persons getting vaccinated early are systematically different from those vaccinated later. Third, important data including SARS-CoV-2 exposure risk and mask use were not collected, which might result in residual confounding. Fourth, the circulating variants in the United States continue to change, and results of this study might not be generalizable to future variants. Fifth, tests used in this study were collected predominantly (although not exclusively) in areas with higher social vulnerability; therefore, data might not be fully representative of the broader U.S. population. Finally, these results might be susceptible to bias because of differences in testing behaviors between vaccinated and unvaccinated persons.

In this study of immunocompetent persons tested at ICATT locations, bivalent booster doses provided significant additional protection against symptomatic SARS-CoV-2 infection during a period when Omicron variant BA.4/BA.5 lineages and their sublineages predominated. All persons should stay up to date with recommended COVID-19 vaccines, including bivalent booster doses, if it has been ≥2 months since their last monovalent vaccine dose (*1*).

**Summary**

**What is already known about this topic?**

Monovalent mRNA COVID-19 vaccines were less effective against symptomatic infection during the period of SARS-CoV-2 Omicron variant predominance.

**What is added by this report?**

In this study of vaccine effectiveness of the U.S.-authorized bivalent mRNA booster formulations, bivalent boosters provided significant additional protection against symptomatic SARS-CoV-2 infection in persons who had previously received 2, 3, or 4 monovalent vaccine doses. Due to waning immunity of monovalent doses, the benefit of the bivalent booster increased with time since receipt of the most recent monovalent vaccine dose.

**What are the implications for public health practice?**

All persons should stay up to date with recommended COVID-19 vaccinations, including bivalent booster doses for eligible persons.

## Footnotes

* https://www.cdc.gov/icatt/index.html

† Social vulnerability index (SVI) is a tool that uses U.S. Census Bureau data on 16 social factors to rank social vulnerability by U.S. Census Bureau tract. The scale is from 0 to 1; higher SVIs represent more vulnerable communities. Tests with missing SVI data (<1% of total) were excluded from all analyses. https://www.atsdr.cdc.gov/placeandhealth/svi/data_documentation_download.html

§ Only month and year of receipt were reported for each vaccine dose from some participating pharmacies; therefore, the number of months between a vaccine dose and testing is a whole number calculated as the difference between the month and year of testing and the month and year of the vaccine dose. Persons reporting an mRNA booster dose on or after September 1, 2022, were assumed to have received a bivalent dose because no monovalent mRNA doses were authorized for use as booster doses at that time. For doses received in the same month or the month before SARS-CoV-2 testing, an additional question was asked to specify whether the dose was received ≥2 weeks before testing, and only doses received ≥2 weeks before testing were included.

¶
https://covid.cdc.gov/covid-data-tracker/#variant-proportions

** Test registration forms asked persons to report if they had an immunocompromising condition and provided the following examples: immunocompromising medications, solid organ or blood stem cell transplant, HIV, or other immunocompromising conditions.

††
https://www.cdc.gov/coronavirus/2019-ncov/symptoms-testing/testing.html

§§ Multivariable logistic regression models were controlled for age, gender, race, ethnicity, SVI of the testing location, underlying conditions (presence versus absence), state of residence of person tested, pharmacy chain conducting the test, local incidence (cases per 100,000 by site zip code during the 7 days preceding test date), and date of testing. The following underlying conditions were included on the survey: heart conditions, high blood pressure, overweight or obesity, diabetes, current or former smoker, kidney failure or end stage renal disease, cirrhosis of the liver, chronic lung disease (such as chronic obstructive pulmonary disease, moderate to severe asthma, cystic fibrosis, or pulmonary embolism).

¶¶ 45 C.F.R. part 46.102(l)(2), 21 C.F.R. part 56; 42 U.S.C. Sect. 241(d); 5 U.S.C. Sect. 552a; 44 U.S.C. Sect. 3501 et seq.

***
https://www.cdc.gov/vaccines/acip/meetings/downloads/slides-2022-09-01/04-COVID-Link-Gelles-508.pdf

††† https://covid.cdc.gov/covid-data-tracker/#vaccinations_vacc-people-onedose-pop-pop18

§§§
https://covid.cdc.gov/covid-data-tracker/#vaccinations_vacc-people-booster-percent-pop5

## References

[1] Rosenblum HG, Wallace M, Godfrey M, Interim recommendations from the Advisory Committee on Immunization Practices for the use of bivalent booster doses of COVID-19 vaccines—United States, October 2022. MMWR Morb Mortal Wkly Rep 2022;71:1436–41. 10.15585/mmwr.mm7145a2 36355612 PMC9707353

[2] Miller MF, Shi M, Motsinger-Reif A, Weinberg CR, Miller JD, Nichols E. Community-based testing sites for SARS-CoV-2—United States, March 2020–November 2021. MMWR Morb Mortal Wkly Rep 2021;70:1706–11. 10.15585/mmwr.mm7049a3 34882655 PMC8659188

[3] Fleming-Dutra KE, Britton A, Shang N, Association of prior BNT162b2 COVID-19 vaccination with symptomatic SARS-CoV-2 infection in children and adolescents during Omicron predominance. JAMA 2022;327:2210–9. 10.1001/jama.2022.7493 35560036 PMC9107063

[4] Britton A, Embi PJ, Levy ME, Effectiveness of COVID-19 mRNA vaccines against COVID-19–associated hospitalizations among immunocompromised adults during SARS-CoV-2 Omicron predominance—VISION Network, 10 states, December 2021–August 2022. MMWR Morb Mortal Wkly Rep 2022;71:1335–42. 10.15585/mmwr.mm7142a4 36264840 PMC9590295

[5] Wang Q, Bowen A, Valdez R, Gherasim C. Antibody responses to Omicron BA.4/BA.5 bivalent mRNA vaccine booster shot. bioRxiv [Preprint posted online October 24, 2022]. https://www.biorxiv.org/content/10.1101/2022.10.22.513349v1 10.1101/2022.10.22.513349

[6] Collier A, Miller J, Hachmann N. Immunogenicity of the BA.5 bivalent mRNA vaccine boosters. bioRxiv [Preprint posted online October 25, 2022]. https://www.biorxiv.org/content/10.1101/2022.10.24.513619v1 10.1101/2022.10.24.513619

[7] Clarke KE, Jones JM, Deng Y, . Seroprevalence of infection-induced SARS-CoV-2 antibodies—United States, September 2021–February 2022. MMWR Morb Mortal Wkly Rep 2022;71:606-8. 10.15585/mmwr.mm7117e3 35482574 PMC9098232

